# *TomoCPT*: a generalizable model for 3D particle detection and localization in cryo-electron tomograms

**DOI:** 10.1107/S2059798325000865

**Published:** 2025-02-01

**Authors:** Pranav N. M. Shah, Ruben Sanchez-Garcia, David I. Stuart

**Affiliations:** a Division of Structural Biology University of Oxford Roosevelt Drive OxfordOX3 7BN United Kingdom; b School of Science and Technology IE University Paseo de la Castellana 259 28046Madrid Spain; National Center of Biotechnology, CSIC, Spain

**Keywords:** cryo-ET, particle picking, subtomogram averaging

## Abstract

Cryo-electron tomography is a rapidly developing field for studying macromolecular complexes in their native environments and has the potential to revolutionize our understanding of protein function. However, fast and accurate identification of particles in cryo-tomograms is challenging and represents a significant bottleneck in downstream processes such as subtomogram averaging. Here, we present *tomoCPT* (*Tomogram Centroid Prediction Tool*), a transformer-based solution that reformulates particle detection as a centroid-prediction task using Gaussian labels. Our approach, which is built upon the SwinUNETR architecture, demonstrates superior performance compared with both conventional binary labelling strategies and template matching. We show that *tomoCPT* effectively generalizes to novel particle types through zero-shot inference and can be significantly enhanced through fine-tuning with limited data. The efficacy of *tomoCPT* is validated using three case studies: apoferritin, achieving a resolution of 3.0 Å compared with 3.3 Å using template matching, SARS-CoV-2 spike proteins on cell surfaces, yielding an 18.3 Å resolution map where template matching proved unsuccessful, and rubisco molecules within carboxysomes, reaching 8.0 Å resolution. These results demonstrate the ability of *tomoCPT* to handle varied scenarios, including densely packed environments and membrane-bound proteins. The implementation of the tool as a command-line program, coupled with its minimal data requirements for fine-tuning, makes it a practical solution for high-throughput cryo-ET data-processing workflows.

## Abstract 2

*TomoCPT* is a generalizable transformer-based 3D particle-picking tool for cryo-tomographic data.

## 1. Introduction

Cryo-electron microscopy (cryo-EM) is a powerful method enabling the visualization of macromolecular complexes at near-atomic resolution. This technique generates detailed Coulomb potential maps by imaging macromolecules embedded in thin vitreous ice slabs. While single-particle analysis (SPA) remains the most popular method for computing 3D macromolecular structures with electrons, cryo-electron tomography (cryo-ET) offers distinct advantages in certain scenarios. Cryo-ET involves the acquisition of multiple projection images of the same specimen at systematically varied tilt angles, allowing the 3D reconstruction of unique cellular landscapes. This approach is particularly advantageous for studying macromolecular complexes in their native context, revealing both their structure and their spatial relationships with surrounding components. Furthermore, cryo-ET coupled with cryo-focused ion beam milling (cryo-FIB) for sample preparation of vitrified biological samples such as cells (Hong *et al.*, 2023 ▸; Lučić *et al.*, 2005 ▸), tissues (Zhang *et al.*, 2021 ▸; Leistner *et al.*, 2023 ▸; Wang *et al.*, 2023 ▸) and whole organisms (Schiøtz *et al.*, 2024 ▸; Nguyen *et al.*, 2024 ▸) represents a powerful tool for linking protein structures with their cellular context, facilitating an integrative understanding of biological phenomena.

The computational workflow for cryo-ET data processing involves several steps that are included in a number of software packages. These steps include motion correction, CTF estimation, tilt-stack generation and alignment and tomogram reconstruction (Turoňová & Wan, 2024 ▸). Other optional steps can be used to enhance the contrast of the tomograms, typically by using filtering processes, including denoising (Frangakis, 2021 ▸) and missing-wedge compensation (Liu *et al.*, 2022 ▸). The detection of particles in tomograms (also known as particle picking) presents significant computational challenges. While there are some template-free approaches, most of the current methods rely on template matching using templates generated for the protein assembly of interest. Template matching is a computationally intensive process that involves the generatation of thousands of template orientations and the computation of similarity scores for every position in the tomogram (Castaño-Díez *et al.*, 2017 ▸; Chaillet *et al.*, 2024 ▸; Wan *et al.*, 2024 ▸; Tegunov *et al.*, 2021 ▸). Recent studies have shown that achieving high-confidence identification requires fine-grain sampling regimes, further escalating computational demands (Cruz-León *et al.*, 2024 ▸; Chaillet *et al.*, 2023 ▸). Among template-free methods, deep-learning models offer a potentially more efficient solution for localizing the particle, with the added advantages of improved generalizability and reusability compared with template matching, with recent advances showing considerable promise (Zeng *et al.*, 2023 ▸; Moebel *et al.*, 2021 ▸; Rice *et al.*, 2023 ▸; de Teresa-Trueba *et al.*, 2023 ▸; Kiewisz *et al.*, 2023 ▸; Liu *et al.*, 2024 ▸; Huang *et al.*, 2024 ▸). Particularly noteworthy is the development of transformer-based models, which have demonstrated significant improvements in various computer vision tasks (Dosovitskiy *et al.*, 2020 ▸).

The transformer architecture, initially designed for natural language processing, has been successfully adapted for image analysis through vision transformers (ViTs; Dosovitskiy *et al.*, 2020 ▸) and their variants (Han *et al.*, 2023 ▸). Building upon these innovations, the Swin transformer architecture introduced a hierarchical structure and shifted-window approach, enabling the efficient processing of large images while maintaining the ability to model spatially distant but semantically related features in a given image (Tang *et al.*, 2021 ▸).

SwinUNETR (He *et al.*, 2023 ▸; Tang *et al.*, 2021 ▸) represents a powerful fusion of Swin transformers and the U-Net architecture (Ronneberger *et al.*, 2015 ▸), which is particularly well suited for medical image-analysis tasks. This model combines the strengths of Swin transformers in capturing complex spatial relationships with the ability of U-Net to generate high-resolution outputs, making it a promising candidate for particle localization in cryo-ET data.

While these advanced architectures have significantly improved the accuracy of semantic segmentation in various domains, the application of these models to particle localization in cryo-ET presents unique challenges where the signal-to-noise ratio (SNR) is thought to be among the lowest among imaging methods and does not exceed a value of 0.1 (Baxter *et al.*, 2009 ▸). In typical semantic segmentation tasks, the network output can be treated as a confidence map of object locations. However, translating these predictions into precise protein localizations requires additional steps.

Traditional object detection in cryo-ET typically aims to identify objects in a given image by predicting a mask around the object. Whilst this approach has value in identifying large-scale morphological features (for example membranes) in a given tomogram, these segmentation masks can be less effective in identifying macromolecular assemblies. Indeed, the best confirmation for an assembly identified by any of the aforementioned methods is through subtomogram averaging and classification steps to differentiate between true-positive and false-positive picks. Conventionally, object localization from predicted segmentation masks involves computer vision operations such as thresholding, morphological operations and non-maximum suppression to assign centroid values to the detected objects. While these post-processing steps can often achieve good-quality results, they are computationally expensive, require extensive fine-tuning and may not generalize well across different data sets or particle types. For instance, there is a risk of excluding particles that are too close together and missing them from the analysis, which is likely to impact subsequent biological inferences.

Considering these disadvantages, we sought to reformulate the problem of particle detection in tomograms as a centroid-detection task. This approach has advantages over a full-object detection task since a strong prior can be placed on the location of the particle centroid during training by way of a Gaussian label that tapers off as a function of distance from the particle centre. This strategy has successfully been used for tasks as varied as cell counting in microscopic images, monitoring crowds in surveillance systems and wildlife censuses (Lempitsky & Zisserman, 2010 ▸).

In this paper, we describe our program *Tomogram Centroid Prediction Tool* (*tomoCPT*), which aims to improve upon classical approaches by directly predicting masks with the centroids of the particles rather than the entire particle volume. We demonstrate that this method is generalizable and improves particle-picking efficiency compared with classical particle binary labelling strategies. It also addresses the limitations of current methods by reducing the need for post-processing steps and enhancing the accuracy of centroid assignment.

## 2. Methods

### 2.1. Label generation for particle centroid annotation

For supervised training of the SwinUNETR-V2 model (He *et al.*, 2023 ▸), pairs of experimental tomograms and label tomograms need to be generated. Because manual annotation of particles is tedious and time-consuming, we aim to ease the process of generating the labelled data by considering only the particle centroids that can be provided in the form of *RELION* STAR files or *IMOD*.mod files.

For our method, training labels for each particle were generated by combining two Gaussians of differing widths: one with a sigma value of 32% of the particle radius (or the longest axis for nonspherical particles) and a second Gaussian with a sigma value of 16% of the particle radius (see Figs. 2*a* and 2*b* for a visual representation of the employed labels). Note that this corresponds to Gaussians of width 95% and 50% of the particle diameter, where width is defined as ±3 times sigma. The summed Gaussian mask aims to more accurately represent the probability density of particle locations compared with binary labels, which cannot encode uncertainty.

Specifically, for each particle *i* of radius *R*, the intensity *I^i^* at any point (*x*, *y*, *z*) is given by
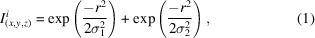
where *r* = (*x*^2^ + *y*^2^ + *z*^2^)^1/2^ is the distance from the centre and σ_1_ and σ_2_ are calculated as
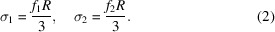
Here, *f*_1_ and *f*_2_ are the fraction of the particle diameter for the first and second Gaussian functions (by default set to 0.95 and 0.5, respectively). The combined *I^i^* is then truncated at radius *R* and normalized as
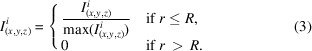
Finally, the label tomogram is obtained by combining the representation of all particles as
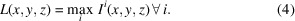


#### 2.1.1. Data pre-processing

Tomograms are resized to the sampling rate at which the particle size is 10 pixels. Then, each tomogram (*T*_input_) is normalized using a robust normalization strategy:
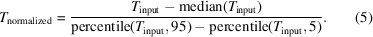
The normalized tomograms are then divided into overlapping cubes of 64 × 64 × 64 pixels using a stride of 32 pixels. To address the class imbalance, all cubes containing at least one positively annotated voxel are retained, while an equal number of negative cubes (containing no positive voxels) are randomly sampled.

### 2.2. Training

The training process spanned at most 150 epochs. To prevent overfitting and unnecessary computational expense, we implemented an early stopping mechanism with a patience of 36 epochs. We processed the data in batches of ten cubes using an initial learning rate of 0.0004. The learning rate was halved when the validation loss did not improve for six consecutive epochs. As the optimizer, we employed AdamW (Loshchilov & Hutter, 2017 ▸) with a weight decay of 1 × 10^−10^.

### 2.3. Loss function

We use a loss function that combines a weighted Huber loss (Huber, 1964 ▸) that operates at the pixel level with a spatial gradient loss that tries to improve edge-detection sensitivity,
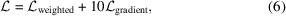
where
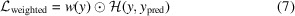
and
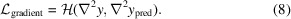
Here, 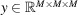 is the label tomogram, 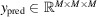 is the predicted centroid tomogram, 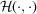 denotes the Huber loss, ⊙ represents element-wise multiplication and ∇^2^ is the second-order derivative operator.

The weight function *w*(*y*) generates a per-voxel weight array
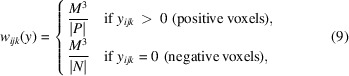
where |*P*| and |*N*| are the number of positive and negative voxels, respectively.

The ∇^2^ operator is implemented using the Kornia function kornia.filters.spatial_gradient3d (Riba *et al.*, 2020 ▸).

### 2.4. Centroid extraction from the predicted labels

The *scikit-image* (van der Walt *et al.*, 2014 ▸) function peak_local_max was directly employed over the predicted tomogram to retrieve the coordinates of the centroids. This function performs a neighbourhood-based search to detect local peaks, which are defined as pixels whose intensity values are higher than those of their neighbours. Specifically, the algorithm compares each pixel with the values in a cubic neighbourhood of size min_distance (by default the particle diameter). Pixels that do not surpass their neighbours within this distance are suppressed, and only the most prominent peak in each neighbourhood is retained. After this, the coordinates of the remaining peaks whose confidence is larger than a user-provided threshold (typically 0.3) are reported as the predicted centroids. We observed that a threshold of 0.3 provided a good balance between false-positive and true-positive picks. However, this value should be chosen by the user on a case-by-case basis following the inspection of confidence maps.

### 2.5. Calculating metrics for particle detection

To evaluate the performance of *tomoCPT* in predicting particle centroids from tomograms of different data sets, we implemented two types of computations: estimates of precision and recall, and estimations of root-mean-squared error of the centroid locations. In both cases the list of predicted coordinates is obtained by applying a given confidence threshold. After this, we associate each ground-truth coordinate with its nearest predicted coordinate using the Jonker–Volgenant algorithm (Jonker & Volgenant, 1988 ▸) with the Euclidean distances matrix as the cost matrix. We note that when working with experimental data there are no real ground-truth coordinates, but for simplicity we consider the coordinates obtained with our processing pipeline (see Section 2.6) as the ground-truth coordinates.

Once the predicted and ground-truth coordinates have been matched, a distance threshold, defined as half the object diameter, was applied to classify the matches. Predicted centroids within this threshold of their corresponding ground-truth points were categorized as true positives, whilst those exceeding this criterion were labelled as false positives. Ground-truth points without a corresponding prediction within the specified threshold were classified as false negatives.

We determined optimal detection thresholds by performing a percentile sweep (2–98% in steps of 8%) on the confidence values of *tomoCPT*-predicted centroids to identify the percentile yielding the highest F1 score (F1_max_) for each particle specimen (Supplementary Fig. S2).

Following this classification, we quantified the predictive performance of the model using standard metrics: precision, recall and F1 score (Sasaki, 2007 ▸; Hicks *et al.*, 2022 ▸). These metrics were calculated as follows:
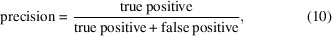

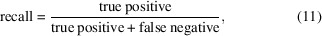

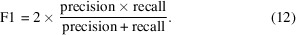
Additionally, we report the root-mean-square error (RMSE) normalized by the particle dimension at F1_max_, which was calculated as
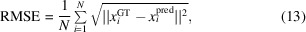
where 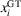 is the ground-truth coordinate matched to the *i*th predicted coordinate 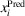 and *N* is the number of ground-truth coordinates or the number of predicted coordinates, whichever is smaller. Only true-positive predicted coordinates were employed in this calculation.

### 2.6. Characteristics of the data sets used for training and validation

The data sets represent a wide range of particles, ranging from 1000 Å (rotavirus) to 135 Å (influenza haemagglutinin; HA). Additionally, the data sets consist of a mixture of tomograms generated from two different software packages, *Warp* (Tegunov & Cramer, 2019 ▸) and *NOVACTF* (Turoňová *et al.*, 2017 ▸), and diverse filtration methods such as CTF correction, CTF-aware Wiener-like filtering (*Warp*) and CNN-based denoising (*Warp*). Together these data represent different SNR levels, contributing to the versatility of *tomoCPT*.

#### 2.6.1. Pre-processing of in-house tilt-series data and particle centroid annotation

The data sets used in the particle-picking case studies were processed in the *Warp* software package (version 1.0.9;Tegunov & Cramer, 2019 ▸). The pre-processing steps consisted of simultaneous 2D motion correction and CTF estimation on individual frames of the tilt series. Due to the limited dose per tilt, a simplified 2 × 2 × *N* (where *N* is the number of frames) motion and CTF model was used. Tilt stacks were then built from the frame-series data utilizing information stored in the microscope metadata files and subjected to *IMOD* tilt-series alignment using either fiducial-based alignment or patch tracking depending on whether or not the images contained fiducials. The aligned parameters were then imported into *WarpTools* and dose-weighted 3D CTF parameters were estimated for the tilt series. Following this step, CTF-corrected tomograms were generated and used for particle centroid annotation.

Coordinates used in a previous subtomogram averaging study of rotavirus assembly intermediates (Shah *et al.*, 2023 ▸) were annotated using Wiener-like filtered tomograms reconstructed at a pixel size of 10 Å per pixel using *crYOLO* (version 1.8.0; Wagner *et al.*, 2019 ▸). In the case of influenza HA spike, *crYOLO* was used to localize centroids in denoised tomograms reconstructed at a pixel size of 15 Å per pixel. Equine rhinitis A virus (ERAV) and Murine norovirus (MuNoV) particle centroids were manually annotated in denoised and Wiener-like filtered tomograms, respectively, sampled at 15 Å per pixel using *IMOD* (version 4.12.64; Mastronarde & Held, 2017 ▸; Kremer *et al.*, 1996 ▸).

Subsequently, subtomograms generated using *Warp* were subjected to 3D classification followed by 3D refinement for particle-pose determination using *RELION* (veresion 3.1.2; Zivanov *et al.*, 2019 ▸). These particle coordinates served as ground-truth data against which the performance of the network was measured.

#### 2.6.2. Publicly available data sets

In addition to the four in-house data sets, we incorporated two publicly available data sets accessed from the Chan–Zuckerberg Cryo-ET Data Portal (Ermel *et al.*, 2024 ▸). Data set ID 10003 consists of tomograms of *Mycobacterium pneumoniae* bacterial cells treated with the ribosome-binding antibiotic chloramphenicol (Tegunov *et al.*, 2021 ▸). The deposited tomograms were CTF-corrected and reconstructed using the *Warp* software package. The raw data were deposited in EMPIAR with accession ID 10731. Data set ID 10006 comprises CTF-corrected tomograms of purified SARS-CoV-2 particles reconstructed with weighted back-projection (WBP) using *NOVACTF* (Ke *et al.*, 2020 ▸). The raw data were deposited in EMPIAR with accession ID 10493. In addition to the tomograms, the data sets include expertly curated ground-truth centroids, which were also obtained from the portal.

#### 2.6.3. Training/validation/testing split of data

During training to ensure robust validation, we allocated 20% of the combined training and validation data for validation purposes. Furthermore, from each particle specimen data set used for training, a randomly chosen subset of five tomograms were included into a test set. The performance of the network for different particle specimens was measured using the performance metrics described in Section 2.5.

#### 2.6.4. Pre-processing of tilt-series data used in case studies

The sata sets used in the case studies were pre-processed as described before, with the exception of using the *WarpTools*/*M* software package, which is the Linux-compatible version of *Warp.*

#### 2.6.5. Centroid identification and subtomogram averaging for apoferritin (EMPIAR 10491)

EMDB entry EMD-11334 was used as a template for identifying apoferritin molecule centroids in tomograms reconstructed at a pixel size of 8 Å per pixel. The angular search was performed on a sphere sampled every 7.5°. Only centroids with peak values of ≥6 were retained for downstream steps of subtomogram averaging and refinement in *M*. In the case of *tomoCPT*, only centroids with confidence values of ≥0.7 were retained. Additionally, points closer than the diameter of the molecule were excluded.

Initial pose estimation was performed on defocus-corrected subtomograms resampled on a 4 Å per pixel grid with a box size of 64 pixels in *RELION* (version 4.0.2; Kimanius *et al.*, 2021 ▸) using gold-standard refinement parameters. The orientations and poses determined from this step served as inputs for *M*, which iteratively optimizes the electron-optical and sample deformation-related parameters in the imaging (forward) mode simultaneously with the particle poses in a supervised manner (Tegunov *et al.*, 2021 ▸).

#### 2.6.6. Centroid identification and subtomogram averaging of SARS-CoV-2 spike

Centroids predicted by *tomoCPT* were used to generate subtomograms at a pixel size of 8 Å per pixel and a box size of 42 pixels. The subtomograms were imported into *RELION* and subjected to an initial round of 3D classification, and classes representing spike molecules were selected and their poses were refined using the gold-standard refinement standards in *RELION* (version 4.0.2; Kimanius *et al.*, 2021 ▸). The initial poses were imported into *M* and subjected to iterative rounds of refinement in which parameters such as stage angles, image and volume warping were refined until no improvement in resolution was observed.

#### 2.6.7. Centroid identification and subtomogram averaging of rubisco (EMPIAR 11125)

To fine-tune the base model for picking rubisco molecules within carboxysomes, *Halothiobacillus neapolitanus* carboxysome tomograms deposited in the Chan–Zuckerberg CryoET Data Portal (Ermel *et al.*, 2024 ▸; accession ID 10021) were used. The tomograms (8.832 Å per pixel) were reconstructed using *NOVACTF*, which uses a WBP algorithm (Radermacher, 1988 ▸) for tomogram reconstruction. A total of five tomograms (lma2019-08-21-1, lma2019-08-21-10, lma2019-08-21-11, lma2019-08-21-2 and lma2019-08-21-6) together with expertly annotated ground-truth coordinates obtained from the authors (Lauren Ann Metskas, personal communication) were used to fine-tune the base model for 20 epochs with a starting learning rate of 0.00005.

To be able to verify the quality of particles identified by *tomoCPT*, subtomogram averaging was performed using the raw data deposited in EMPIAR under accession ID 11125 (Metskas, Ortega *et al.*, 2022 ▸). This entry consists of three tilt series with raw frame-series data (CB_02, CB_29 and CB_59) and is a subset of the data deposited in the CryoET Data Portal. The raw data were pre-processed as described in the preceding sections, and *tomoCPT* with fine-tuned weights was used to predict rubisco centroids from CTF-corrected tomograms sampled on an 8 Å per pixel grid. Particle centroids with a probability score of less than 0.4 were excluded from the subtomogram-averaging analysis.

## 3. Results

### 3.1. Software design and workflow

*TomoCPT* is a user-friendly command-line program written in Python to facilitate the automatic extraction of particle centroids from cryo-electron tomograms. The standard use case involves three key steps.

**Figure 1:**
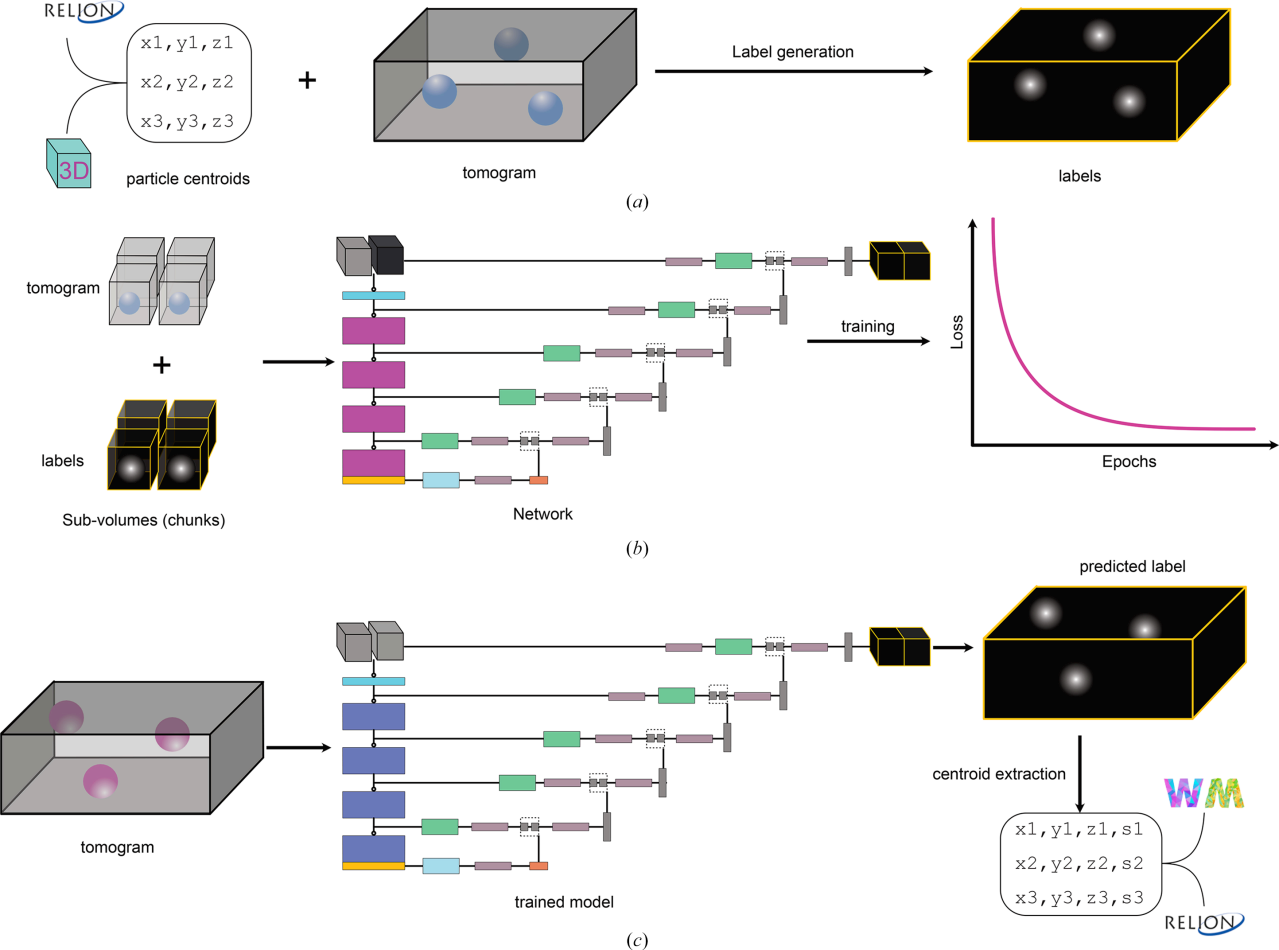
*TomoCPT* design and workflow. The program consists of three main steps. (*a*) Tomogram–label pair generation using centroids provided either in the form of *RELION* STAR files or *IMOD* model files. (*b*) Tomogram resizing followed by data chunking and training until the model converges or training loss does not improve for 36 consecutive epochs. (*c*) Centroid prediction with the trained model; centroids are stored in the form of a STAR file.

(i) Generation of label volume pairs using centroids from *RELION*-formatted STAR files (Hall, 1991 ▸) or *IMOD* model files (Kremer *et al.*, 1996 ▸) for manually generated centroids (Fig. 1 ▸*a*).
(ii) Training of the network with inputs that are normalized and rescaled such that the target particle radius (or longest axis dimension) is large enough to be enclosed within a single chunk. In our experiments we used a chunk size of 64 pixels and a particle radius of 10 pixels (we note that altering this parameter had an insignificant effect on centroid detection; Supplementary Fig. S1), followed by an overlapping data-chunking strategy to manage memory constraints (see Section 2). The network underpinning *tomoCPT* is the SwinUNETR-V2 architecture (He *et al.*, 2023 ▸), implemented in the MONAI framework (Cardoso *et al.*, 2022 ▸), and has been shown to be particularly effective for medical image-segmentation tasks. We leverage the capabilities of the PyTorch Lightning framework (Falcon *et al.*, 2020 ▸) to perform multi-GPU training and monitor a custom loss function (equation 6) for convergence (Fig. 1 ▸*b*). The training step outputs the best-scoring checkpoint file that can either be used for inference or used for further fine-tuning with new data.
(iii) Finally, at inference time, the input tomograms are rescaled and passed through the network, resulting in a confidence map of centroids. After this, centroid extraction is performed using a user-defined confidence threshold and the shortest allowed nearest-neighbour distance. Predicted centroids are stored in the form of a STAR file which is amenable for use with downstream software packages such as *Warp* (Tegunov & Cramer, 2019 ▸) and *RELION* (Scheres, 2012 ▸) (Fig. 1 ▸*c*).

### 3.2. Gaussian labels enable more precise detection of particle centroids compared with binary labels

The development of our approach for training a neural network to predict particle centroids was significantly influenced by the cross-correlation maps typically generated in conventional template-matching programs used to localize particles in tomograms (Tegunov & Cramer, 2019 ▸; Castaño-Díez *et al.*, 2017 ▸; Wan *et al.*, 2024 ▸; Cruz-León *et al.*, 2024 ▸; Chaillet *et al.*, 2024 ▸). Cross-correlation maps record regions of high similarity between the template and the local subvolumes and are represented as intensity maxima. A peak-finding procedure is subsequently employed to extract centroids and orientations from the highest-scoring voxels, utilizing a user-defined threshold and inter-peak distance.

**Figure 2:**
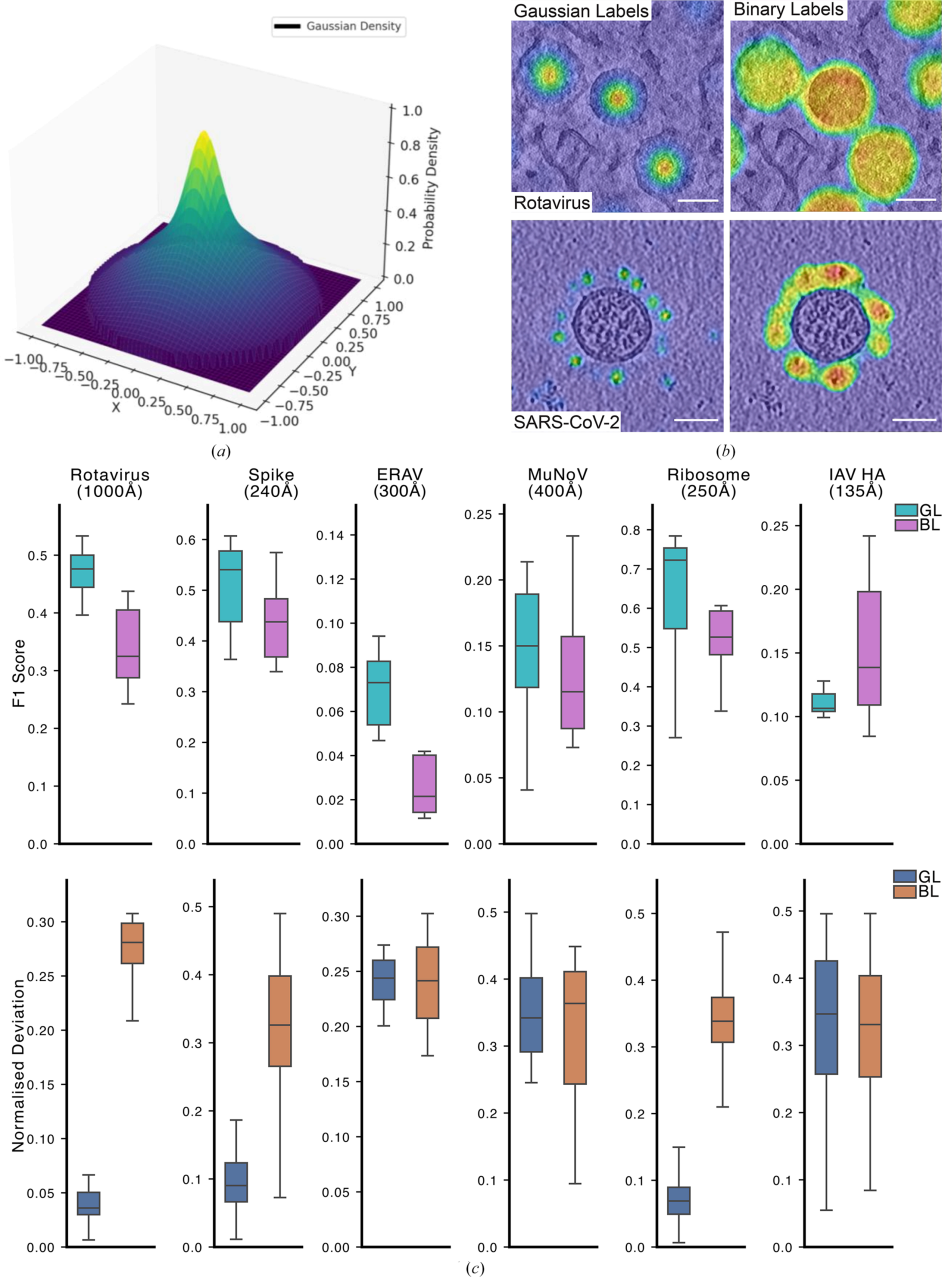
Comparing the Gaussian label approach with the binary label approach for centroid annotation. (*a*) 2D representation of Gaussian labels used to annotate the particle centroids. (*b*) Heatmap of predicted labels using Gaussian (left) and binary (right) labels on rotavirus particles and SARS-CoV-2 spike particles. The scale bar is 100 nm. (*c*) Top: a boxplot of F1 score plotted at the percentile score that yielded its highest value. Cyan bars represent Gaussian labels and pink bars represent Gaussian labels. Bottom: the RMSE deviation as a ratio of particle size is depicted. Blue bars represent Gaussian labels (GL); orange bars represent binary labels (BL).

Inspired by these cross-correlation maps, we designed our training labels as Gaussian probability density functions that are characterized by a sharp peak at the particle centroid and a gradual fall-off (equation 1; Fig. 2 ▸*a*). Next, we evaluated the efficacy of Gaussian labels for centroid prediction using six distinct data sets, namely rotavirus (1000 Å), Equine rhinitis A virus (ERAV; 300 Å), Murine norovirus (MuNoV; 400 Å), ribosomes (250 Å), SARS-CoV-2 spike (240 Å) and influenza virus haemagglutinin (HA; 135 Å), which were either acquired in-house or accessed from public repositories (Table 1 ▸).

A comparative analysis of Gaussian labels versus binary labels reveals superior performance, as demonstrated by the overlay heat maps of prediction tomograms (Fig. 2 ▸*b* and Supplementary Fig. S2). The heat maps show enhanced localization of particle centroids with Gaussian labels, as shown by a more focused and accurate overlay on the viral components.

For quantitative performance assessment across diverse biological specimens, we computed F1 scores, a harmonic mean of precision (equation 10) and recall (equation 11), defined by equation (12) (Sasaki, 2007 ▸; Hicks *et al.*, 2022 ▸). The F1 score ranges from 0 to 1, with 1 being the best possible value.

Analysis of the results (Fig. 2 ▸*c*, top row) shows that particles detected using Gaussian labels achieve consistently higher F1 scores compared with binary labels across all particle types but one. This superior performance extends to the accuracy of centroid assignments for particles identified in the test-set tomograms (Fig. 2 ▸*c*, bottom row). These results establish the robustness and versatility of the Gaussian label approach for accurate particle centroid detection across diverse biological structures in cryo-electron tomography. In the next sections, we refer to the ‘base model’ as the model weights that have been obtained by training our model with the data from these six data sets.

### 3.3. Model evaluation: zero-shot inference versus incremental fine-tuning

Zero-shot inference is an evaluation mode in which the model predicts instances of data classes that are not present during the model-training steps. Following our experiments comparing the suitability of using Gaussian labels for centroid assignment, we sought to assess the ability of *tomoCPT* to predict the centroids of particles that it had not encountered during training. To this end, we used an apoferritin data set (EMPIAR 10491; Tegunov *et al.*, 2021 ▸) and compared the performance of *tomoCPT* against ground-truth coordinates that were assigned using template matching and refined using subtomogram averaging (see Section 2).

**Figure 3:**
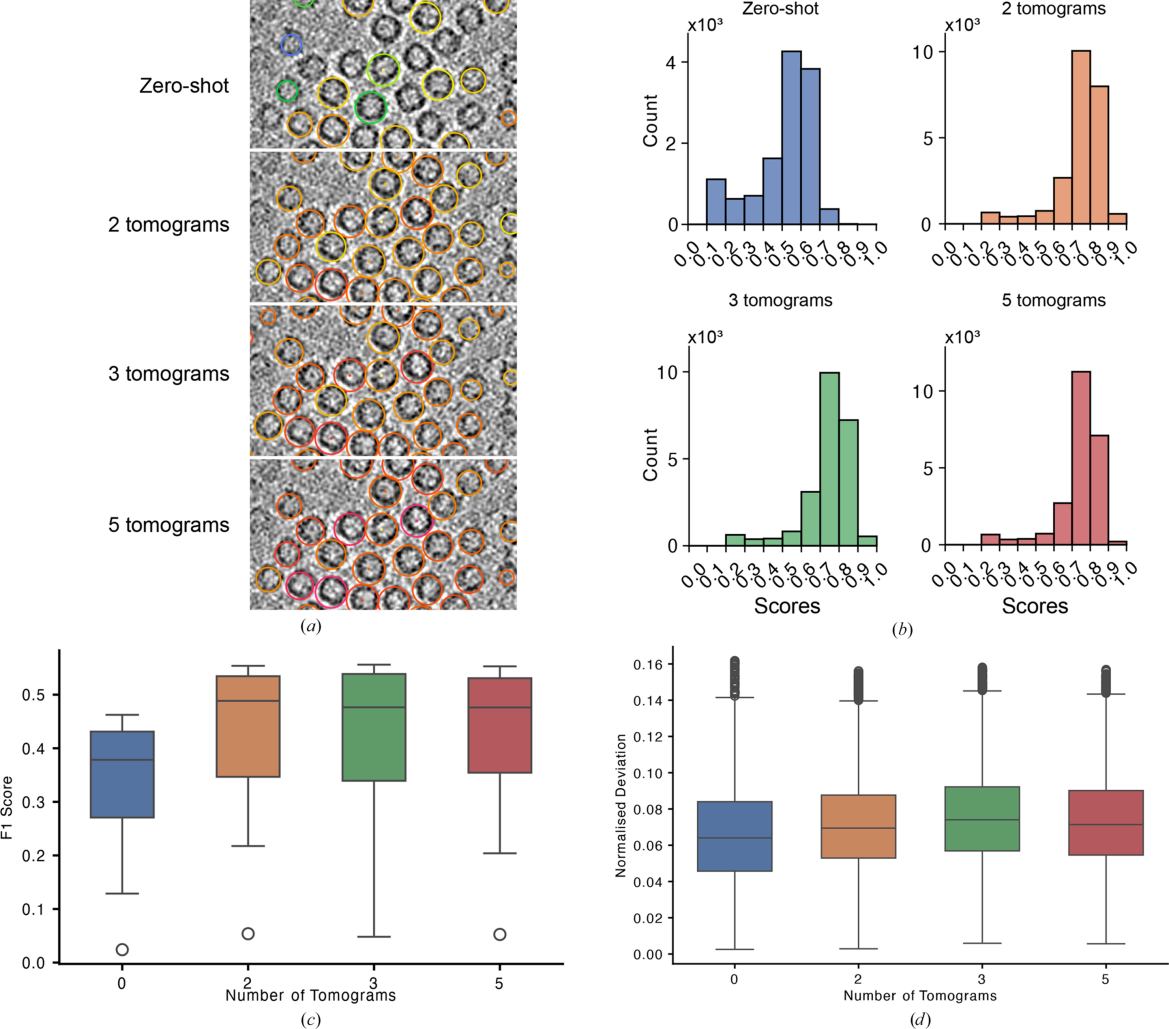
Comparing the performance of *tomoCPT*. (*a*) A section through a tomogram of apoferritin particles with centroids predicted by the model without any fine-tuning and with fine-tuning with two, three and five tomograms. The contours encircling the apoferritin molecules are coloured by the confidence score attributed to that centroid by *tomoCPT*, with cool colours representing low confidence and warmer colours representing high confidence. The scale bar is 25 nm. (*b*) Histograms plotting the distribution of the confidence scores attributed by *tomoCPT*. (*c*) A boxplot of F1 score plotted at the percentile score that yielded its highest value when using the base model and the base model fine-tuned with two, three and five tomograms. (*d*) The RMSE deviation at the maximum F1 score as a ratio of particle size using the base model and the base model fine-tuned with two, three and five tomograms.

From inspection of Fig. 3 ▸(*a*), it is apparent that the *tomoCPT* base model generalizes well enough to assign centroids to individual apoferritin molecules, albeit with low confidence (Fig. 3 ▸*b*). To determine the amount of data that are required to improve the ability of *tomoCPT* to assign centroids to this data set, we conducted a series of fine-tuning experiments using two, three and five tomograms of apo­ferritin data, which correspond to 849, 1001 and 1850 particles, respectively. With just two tomograms for fine-tuning, the F1 score improved; however, F1 did not significantly increase further as we increased the number of tomograms to three and then five (Fig. 3 ▸*c*, Supplementary Fig. S4). We note that in all cases the median accuracy of the networks in predicting the centroids of the apoferritin molecules was within 6% of the accuracy of the diameter of the molecule (Fig. 3 ▸*d*). Importantly, we note that using as few as two tomograms for fine-tuning may be sufficient for robust centroid assignments. Taken together, we show that *tomoCPT* generalizes well enough to identify bona fide particle coordinates, but its performance improves significantly when trained with limited amounts of data.

### 3.4. Centroid prediction and subtomogram averaging of apoferritin

**Figure 4:**
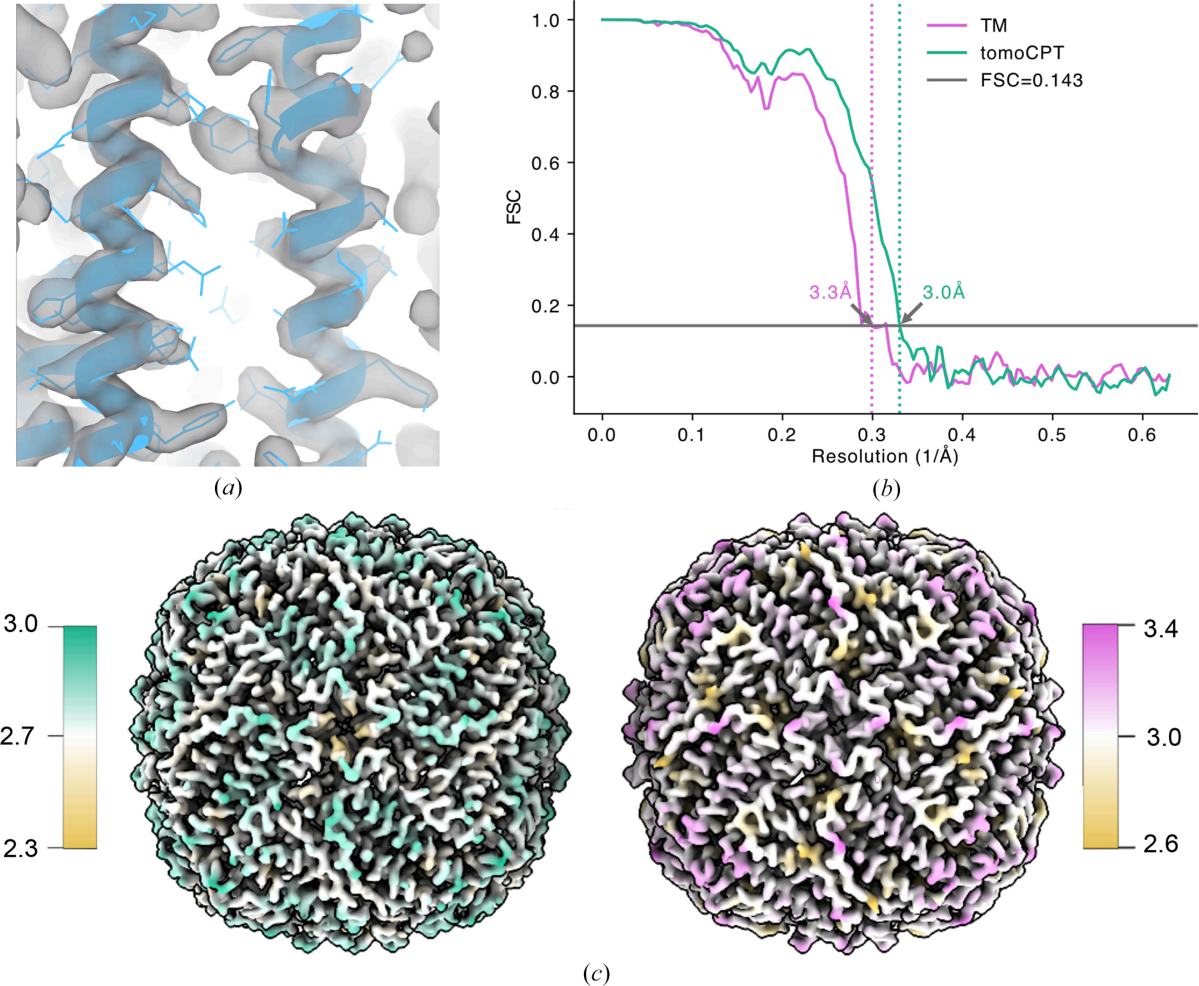
Subtomogram averaging of apoferritin. (*a*) Fit of human apoferritin coordinates (PDB entry 6zsu) in the Coulomb potential map derived from particles predicted by *tomoCPT*. (*b*) Fourier shell correlation (FSC) curves comparing the resolution of maps derived from using *tomoCPT* (green) and template matching (pink). (*c*) Isosurface renderings of the Coulomb potential maps coloured by local resolution using *tomoCPT* (left) and template matching (right).

To assess the quality of centroid predictions by *tomoCPT*, we performed subtomogram averaging on high-confidence centroids (probability score ≥ 0.7) predicted by our best-performing model (Figs. 3 ▸*c* and 3 ▸*d*). This yielded 3931 particles across all tomograms. For a direct comparison, we processed an identical number of centroids identified by template matching, maintaining the same per-tomogram particle distribution as *tomoCPT* (Supplementary Table S1). The resulting subtomograms were processed identically using the *Warp*–*RELION*–*M* pipeline using a developer-provided script (https://github.com/warpem/warp/blob/main/scripts/EMPIAR-10491_5TS_e2e.sh). Subtomograms that were generated using *tomoCPT*-predicted centroids yielded a map with a mean global resolution of 3.03 Å (Figs. 4 ▸*a* and 4 ▸*b*), whereas using subtomograms identified by template matching yielded a map with a mean resolution of 3.34 Å (Fig. 4 ▸*b*).

Furthermore, the average calculated from the *tomoCPT*-predicted centroids demonstrates better local resolution, ranging from 2.3 to 3.0 Å, compared with the TM-derived average, which spans 2.6–3.4 Å (Fig. 4 ▸*c*). The improvement in the maps is further shown by a lower global *B* factor (−41 Å^2^) for the *tomoCPT*-derived map compared with the template-matching-derived map, which required a higher global *B* factor (−66 Å^2^). Together, these results demonstrate that the *tomoCPT*-based centroid-prediction approach not only equals but can surpass the performance of traditional template matching in identifying particles for subtomogram averaging, at least for the data sets tested in this manuscript. The improved resolution and quality of the resulting structures underscore the potential of our method to enhance structure-determination workflows in cryo-electron tomography studies.

### 3.5. Centroid prediction and subtomogram averaging of ChAdOx1 COVID vaccine-expressed SARS-CoV-2 spike molecules

We next evaluated the ability of *tomoCPT* to predict particle centroids from a significantly more difficult and medically relevant data set of SARS-CoV-2 spike proteins transiently expressed on the surface of U2OS cells and resolved to 9 Å resolution from 29 972 particles using a combination of template matching using *emClarity* (Himes & Zhang, 2018 ▸) and extensive manual cleaning (Ni *et al.*, 2023 ▸).

This data set presents challenges for particle picking as the spike molecules are densely clustered and are surrounded by cellular components and other nonspecific contaminants. A conventional method for tackling such a task involves manually tracing the membrane surface through different sections of the tomograms, oversampling the surface and then discarding false-positive picks through multiple rounds of 3D classification procedures. However, this strategy is impractical for large data sets.

**Figure 5:**
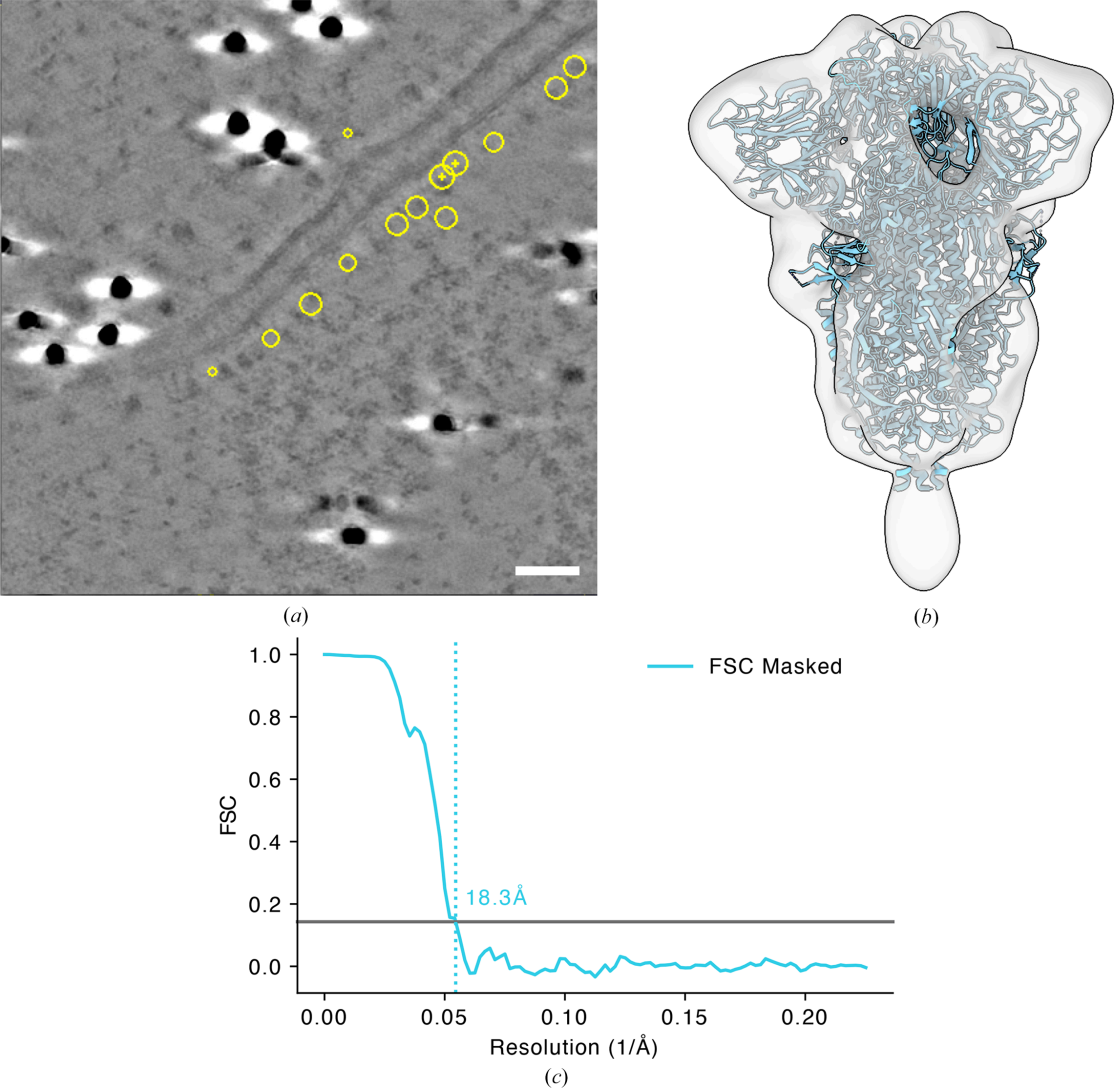
Subtomogram averaging of SARS-CoV-2 spike. (*a*) A section through a tomogram of a cell expressing SARS-CoV-2 spike molecules. Centroids, predicted by *tomoCPT* are highlighted. The scale bar is 25 nm. (*b*) Fit of PDB entry 6vxx in the Coulomb potential map of SARS-CoV-2 spike derived using coordinates predicted by *tomoCPT*. (*c*) FSC trace of the map in (*b*).

Approximately 600 spike centroids from 11 denoised tomograms were manually annotated using the *IMOD* package (Mastronarde & Held, 2017 ▸) and centroid labels were generated. The tomogram–label pairs were then used to fine-tune the base model (described earlier) and used to predict spike centroids from all tomograms. To minimize the extraction of false-positive picks from the tomograms, we implemented an additional feature in *tomoCPT* that enables users to provide a mask for targeted centroid extraction (Supplementary Fig. S5). A total of 3400 particles were subjected to 3D classification in *RELION* (version 4.0.2; Kimanius *et al.*, 2021 ▸), from which 1700 bona fide spike molecules were selected and subjected to gold-standard 3D refinement in *RELION*. This process yielded a map resolved to 18.3 Å resolution (Figs. 5 ▸*b* and 5 ▸*c*) and is reasonably congruent with the atomic model fitted into the density.

Taken together, we show that *tomoCPT* can successfully identify particles in challenging environments and enable the calculation of Coulomb potential maps at mesoscale resolution with a fraction of the data and minimal user involvement.

### 3.6. Centroid prediction of rubisco molecules in carboxysomes

Small object detection in crowded environments remains a challenge for object localization-type tasks. We reasoned that our approach of using Gaussian labels could be particularly suited for identifying particle centroids in such environments.

To this end, we tested our approach on a publicly available data set of rubisco molecules enclosed within polyhedral carboxysome shells (Metskas, Ortega *et al.*, 2022 ▸). These micro-compartments have a mean diameter of 120 nm and can enclose up to 250 rubisco molecules (Ni *et al.*, 2022 ▸; Metskas, Ortega *et al.*, 2022 ▸).

Metskas and coworkers used a combination of geometric picking by manually segmenting individual carboxysome shells and generating an oversampled 3D grid of points from which subtomograms were extracted and extensively classified to yield a map resolved at 4.5 Å using 32 930 particles (Metskas, Wilfong *et al.*, 2022 ▸; Metskas, Ortega *et al.*, 2022 ▸).

**Figure 6:**
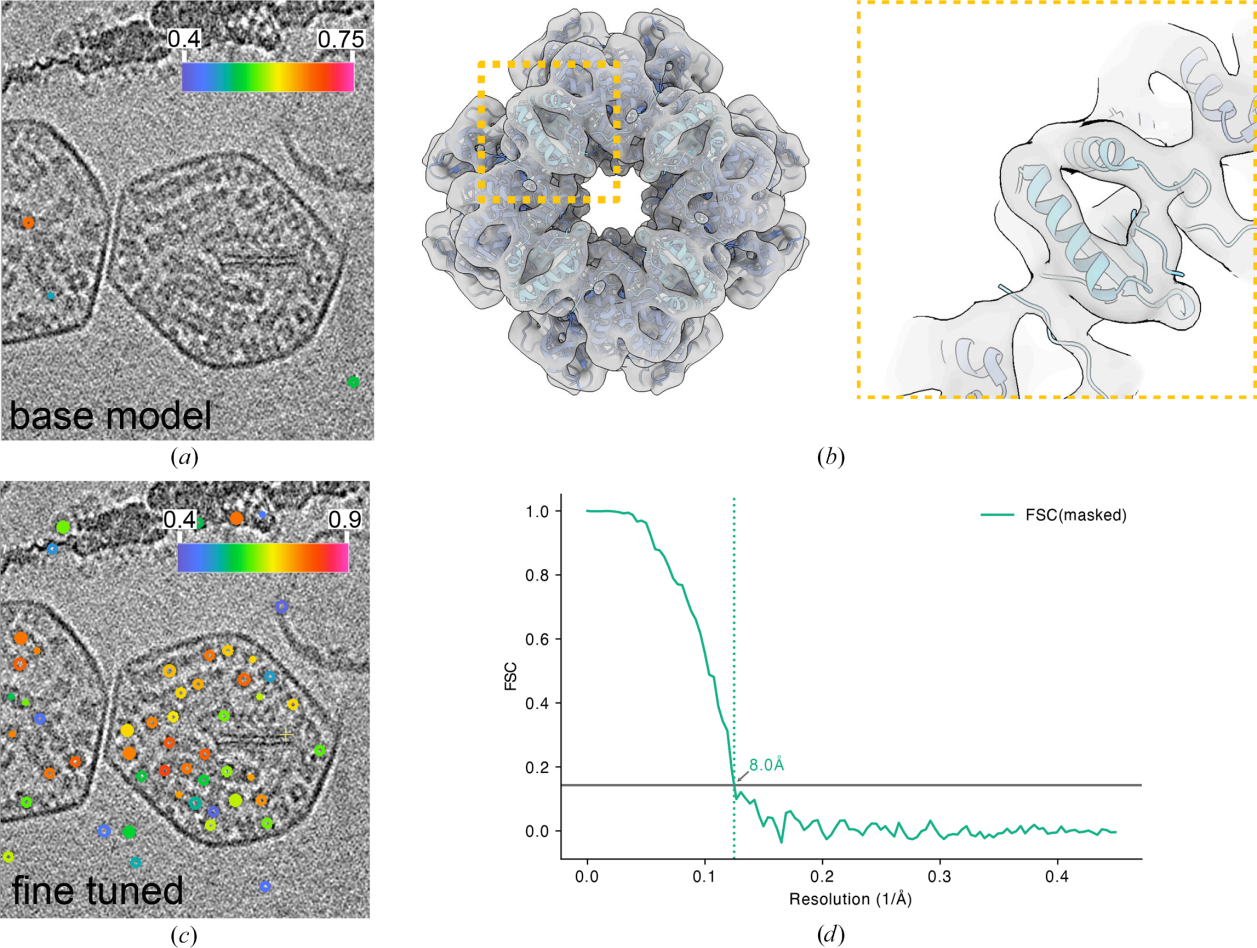
Subtomogram averaging of rubisco molecules. A slice through a tomogram of rubisco-containing carboxysomes with centroids identified by the *tomoCPT* base model (*a*) and fine-tuned on a subset of data (*b*). The contours encircling the rubisco molecules are coloured by the confidence score attributed to that centroid by *tomoCPT*, with cool colours representing low confidence and warmer colours representing high confidence. (*a*) Fit of PDB entry 7zc1 into the Coulomb potential map derived from coordinates identified by the model fine-tuned on rubisco. (*b*) FSC trace of the map shown in (*c*).

In an initial inference run using the base model without any fine-tuning, only 420 rubisco particle centroids were identified (confidence threshold of 0.3); however, the efficiency of detection was low as the base model had not been trained on points as closely clustered as the rubisco molecules (Fig. 6 ▸*a*), thus requiring a further fine-tuning step. Fig. 6 ▸(*b*) shows centroids coloured by the probability score attributed by *tomoCPT*, showing that rubisco particles within the carboxysomes have higher scores than false-positive centroids that lie outside it. Subtomograms extracted from these centroids were then extracted and subjected to a round of 3D classification, which enabled the identification of 1452 bona fide particles whose pose parameters were refined using *RELION* following further refinement in *M*, which yielded a map with a mean resolution of 8.0 Å (Figs. 6 ▸*c* and 6 ▸*d*). This shows that *tomoCPT* can identify particle centroids in crowded environments that can yield subtomogram averages with subnanometre resolution.

## 4. Discussion

Recent advancements in data-collection (Eisenstein *et al.*, 2023 ▸, 2024 ▸; Khavnekar *et al.*, 2023 ▸; Liu *et al.*, 2023 ▸) and sample-preparation techniques (Berger *et al.*, 2023 ▸) in cryo-electron tomography have significantly increased data throughput, enabling the collection of hundreds of tilt series in a single 24 h session. In this study, we have developed *tomoCPT*, a deep-learning tool that predicts particle centroids in cryo-tomograms using a transformer-based network trained on Gaussian labels around centroids (Figs. 1 ▸*a*, 1 ▸*b* and 1 ▸*c*).

Several particle-picking tools utilizing various network architectures have been described in the literature (Zeng *et al.*, 2023 ▸; Moebel *et al.*, 2021 ▸; Rice *et al.*, 2023 ▸; de Teresa-Trueba *et al.*, 2023 ▸; Kiewisz *et al.*, 2023 ▸; Liu *et al.*, 2024 ▸; Huang *et al.*, 2024 ▸). Notably, *DeepPicker* demonstrated that the use of weak labels for annotations and predictions instead of binary labels is superior in performance (Liu *et al.*, 2024 ▸) and outperforms other state-of-the-art particle-picking tools. Similarly, our results demonstrate that the use of Gaussian labels for centroid prediction offers superior performance compared with both binary labels and template matching across a diverse range of biological specimens (Figs. 2 ▸*b* and 2 ▸*c* and Supplementary Figs. S2 and S3) as shown by the higher F1 scores. Furthermore, the use of Gaussian labels enables a precise representation of particle positions (Fig. 2 ▸*a*), eliminating the need for additional post-processing steps to extract particle centroids.

A key advantage of deep-learning methods for object localization is their generalizability to previously unseen data. We demonstrate that *tomoCPT* generalizes effectively to novel data types, as shown by its performance on benchmark data (Figs. 3 ▸*a*–3 ▸*d*, Supplementary Fig. S3). While the base model showed promising results, we observed that fine-tuning with as few as two tomograms was sufficient to achieve a significant increase in performance (Figs. 3 ▸*a*–3 ▸*d*, Supplementary Fig. S3), although with real-world data the target heterogeneity and target counts per tomogram will impact the amount of data required to fine-tune the network for optimal performance. This finding has important implications for the practical application of *tomoCPT*, as it suggests that the model can be rapidly adapted to new protein structures with limited amounts of data.

We assessed the impact of *tomoCPT*-predicted centroids on downstream subtomogram averaging analysis. Using the benchmark apoferritin data set, we observed that the predicted centroids yielded a higher resolution map compared with an equal number of particles identified through template matching (Figs. 4 ▸*a*–4 ▸*c*). This suggests that *tomoCPT* may be more effective at identifying high-quality particles for structural analysis. We also demonstrate the ability of *tomoCPT*to predict coordinates in real-world data sets, such as SARS-CoV-2 spike proteins expressed on cell surfaces (Fig. 5 ▸ and Supplementary Fig. S4) and rubisco molecules enclosed within carboxysomes (Fig. 6 ▸), in both cases yielding particles suitable for subtomogram averaging.

While *tomoCPT* has shown promising results across various data sets, it is important to acknowledge its current limitations.

(i) The formulation of object identification in cryo-tomograms as a centroid-prediction problem means that *tomoCPT* currently lacks a mechanism to pick filamentous assemblies such as microtubules and actin.
(ii) The network was trained on data with objects of uniform size and its capability to simultaneously annotate centroids for differently sized particles in the same field of view remains to be tested.

Self-supervised learning is a powerful paradigm within the deep-learning field, aiming to learn the underlying representation of data to establish a foundation model that would reduce the need for supervised training. This aspect of deep learning in centroid prediction remains unexplored, as insufficient data were available at the time of this study. However, with the increasing availability of curated data in publicly accessible resources such as the Chan–Zuckerberg CryoET Data Portal (Ermel *et al.*, 2024 ▸), the development of a foundation model for cryo-ET data annotation will be a focus of future research efforts.

The development of *tomoCPT* represents a significant step towards more automated and efficient processing of cryo-ET data. By reducing the need for manual intervention and improving the accuracy of particle picking, our method has the potential to accelerate structural studies of diverse biological systems from individual protein complexes to intricate cellular landscapes. Future work will focus on expanding the capabilities of *tomoCPT* to handle a wider range of biological structures and integrating it more seamlessly into existing cryo-ET data-processing pipelines.

## 5. Code availability

The program can be accessed online at https://github.com/shahpnmlab/tomocpt, where instructions to install and run the program are available. Weights will be periodically updated and made available via Zenodo.

## Supplementary Material

Supplementary Figures and Table. DOI: 10.1107/S2059798325000865/sor5001sup1.pdf

## Figures and Tables

**Table 1:** Data sets used in the study

	No. of particles		No. of tomograms			
	Training set	Test set	Training set	Test set	Accession ID	Reference
Data set 1						
Rotavirus	701	23	51	5	In-house	Shah *et al.* (2023 ▸)
SARS-CoV-2 spike	1098	227	40	5	EMPIAR 10493	Ke *et al.* (2020 ▸)
Equine rhinitis A virus	108	22	8	5	In-house	Unpublished
Murine norovirus	335	42	19	5	In-house	Unpublished
*M. pneumoniae* ribosomes	3292	1271	10	5	EMPIAR 10731	Tegunov *et al.* (2021 ▸)
Influenza virus HA	1629	171	15	5	In-house	Unpublished
Data set 2						
Apoferritin_2	849	—	2	—	EMPIAR 10248	Tegunov *et al.* (2021 ▸)
Apoferritin_3	1001	—	3	—	EMPIAR 10248	Tegunov *et al.* (2021 ▸)
Apoferritin_5	1850	—	5	—	EMPIAR 10248	Tegunov *et al.* (2021 ▸)
Data set 3						
ChAdOx1 SARS-CoV-2 spike	667	—	11	—	In-house	Ni *et al.* (2023 ▸)
Data set 4						
Rubisco	3070	—	5	—	DS-10021/EMPIAR 11125	Metskas, Ortega *et al.* (2022 ▸)
