# Supplementary material for: TomoCPT: a generalizable model for 3D particle detection and localization in cryo-electron tomograms
Source: Acta Crystallogr D Struct Biol. 2025 Feb 1;81(Pt 2):63–76. doi: 10.1107/S2059798325000865 (PMC11804251; doi:10.1107/S2059798325000865)
Supplement: Supplementary file 1 [file d-81-00063-sup1.pdf]

STRUCTURAL BIOLOGY

Volume 81 (2025)

Supporting information for article:

TomoCPT: a generalizable model for 3D particle detection and localization in cryo-electron tomograms

Pranav N. M. Shah, Ruben Sanchez-Garcia and David I. Stuart

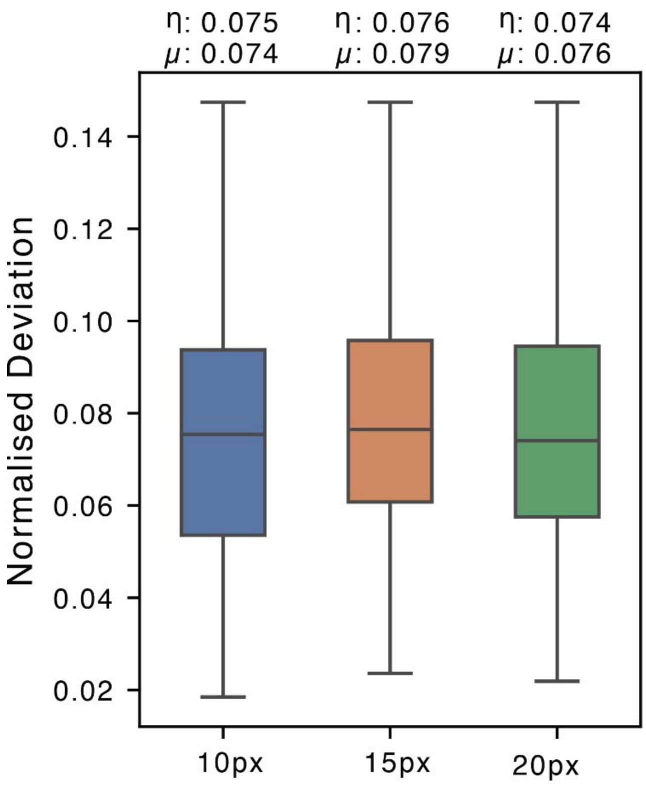

Figure S1 Effect of varying the particle size on the networks ability to detect particle centroids. The RMSE deviation as a ratio of particle size is depicted (μ=mean deviation and η=median deviation).

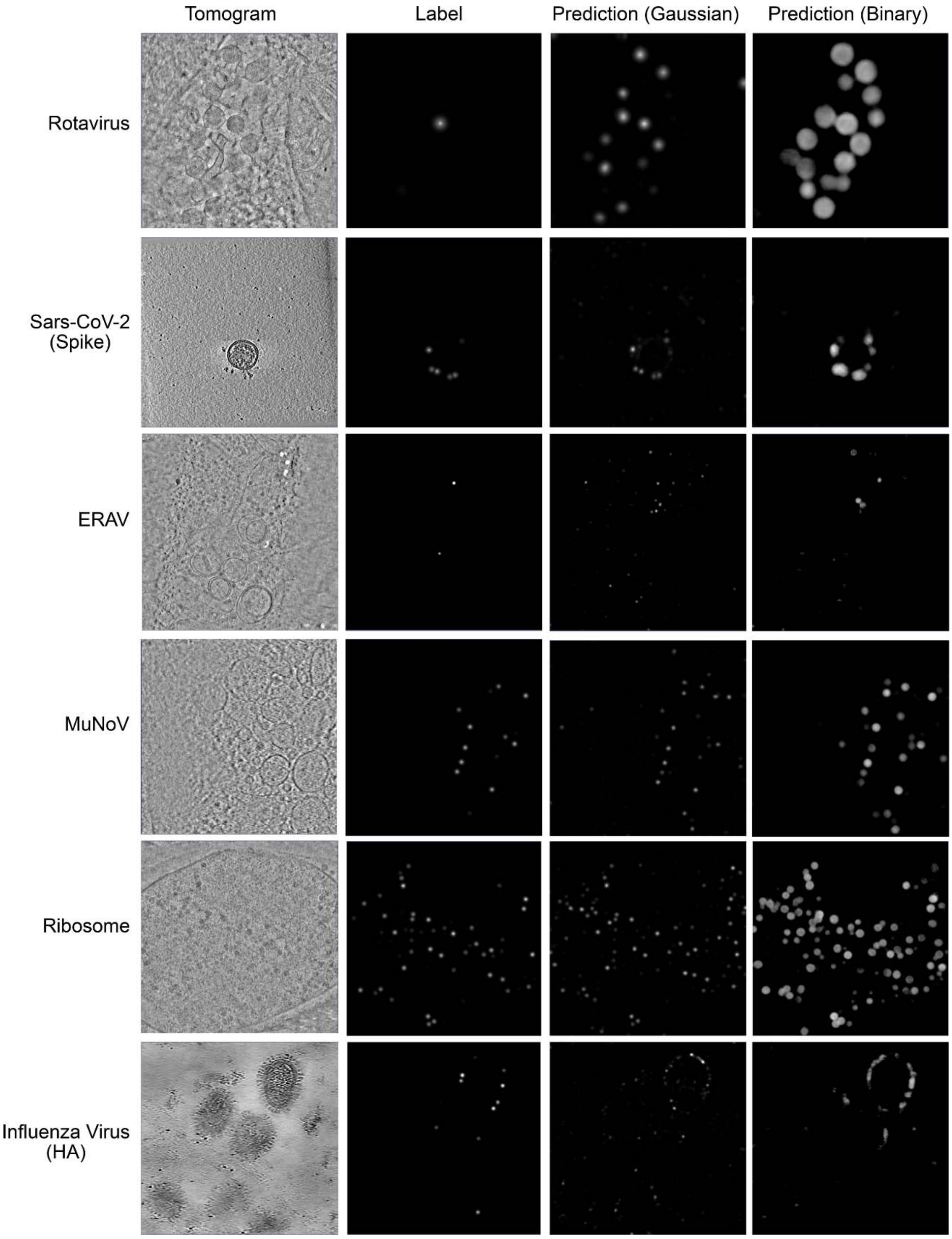

Figure S2 Sections through individual tomograms for the different particle specimens are presented with the ground truth labels as well as the predicted results using both gaussian labels as well as binary labels.

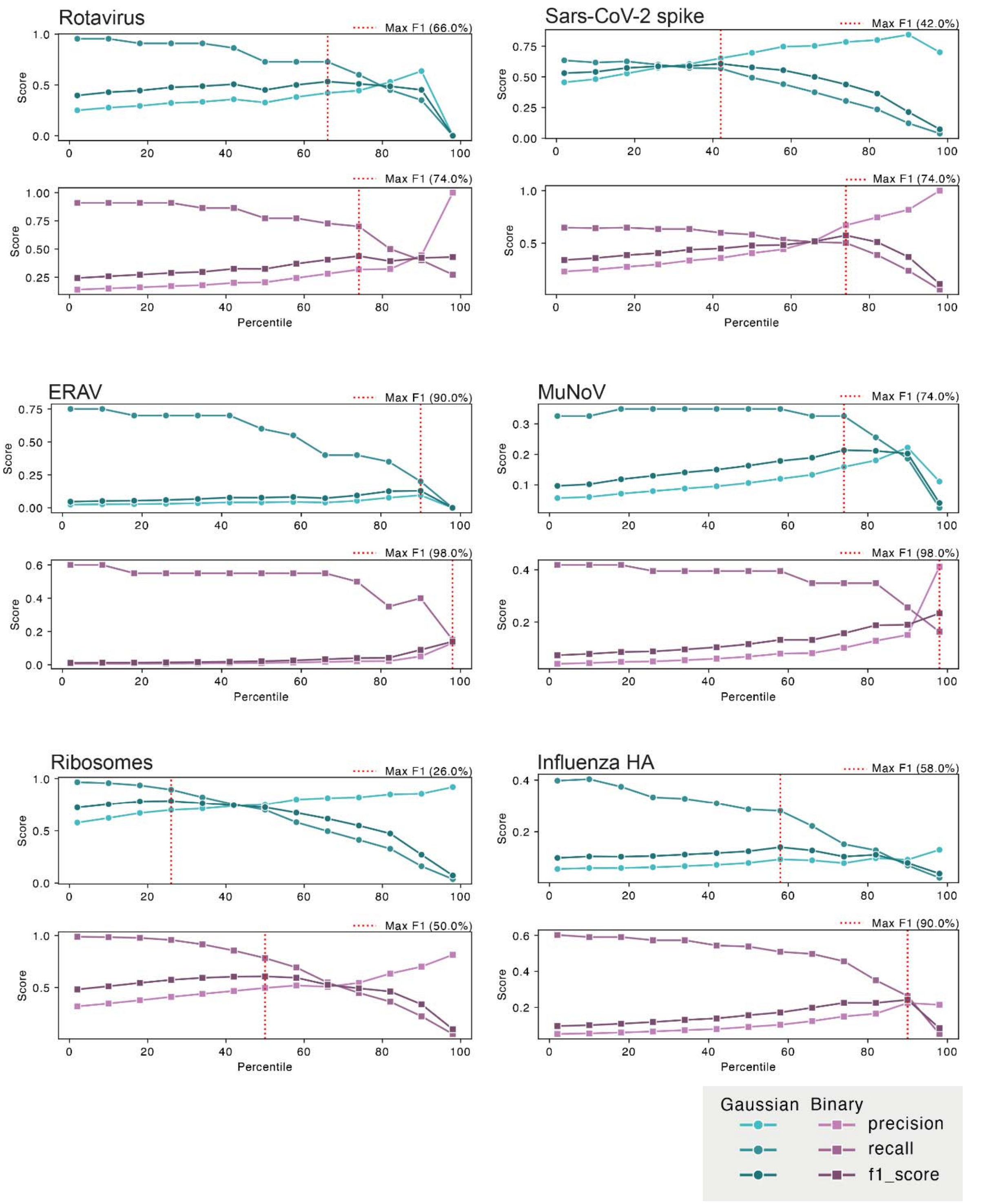

Figure S3 Precision, recall and F1 score for the different particle specimens used to train the base model and described in Figure 1 are plotted against different threshold values. The point of maximum F1 score is highlighted with a red-dotted line.

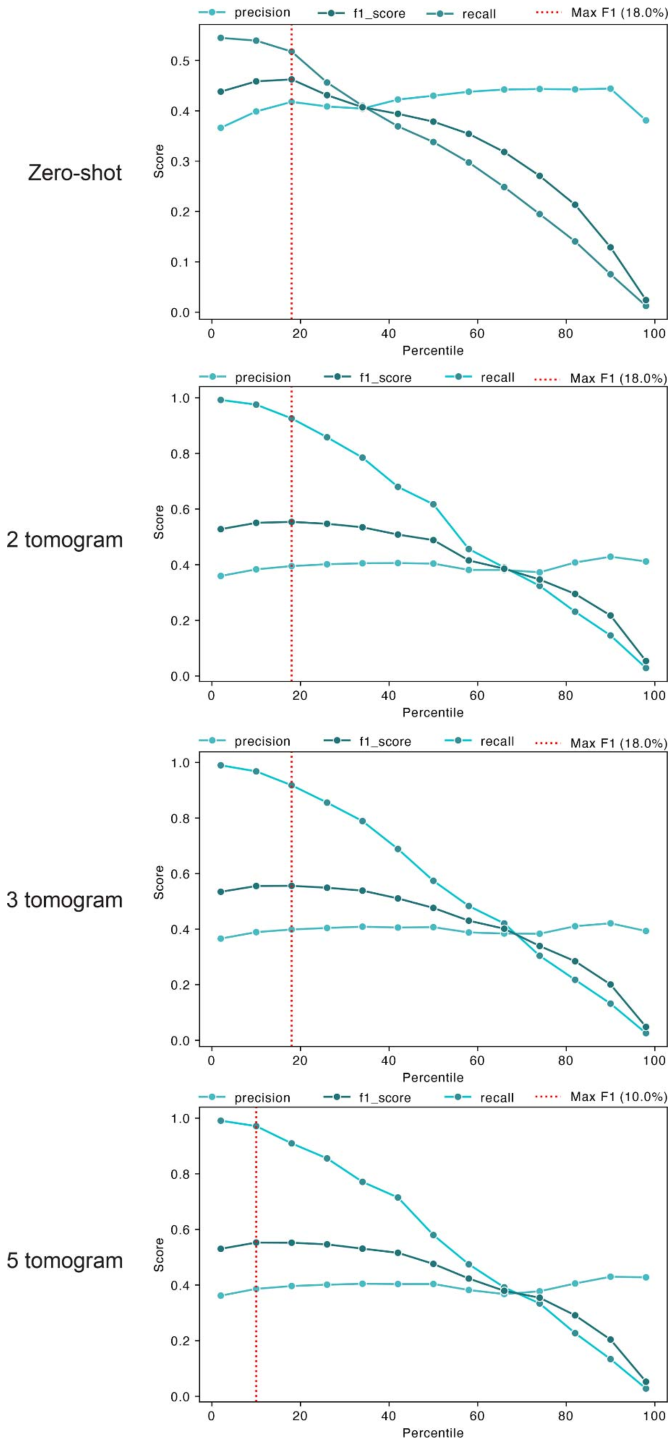

Figure S4 Precision, recall and F1 score plotted against threshold values for training the base model and the base-model finetuned with 2,3, and 5 tomograms. The point of maximum F1 score is highlighted with a red-dotted line.

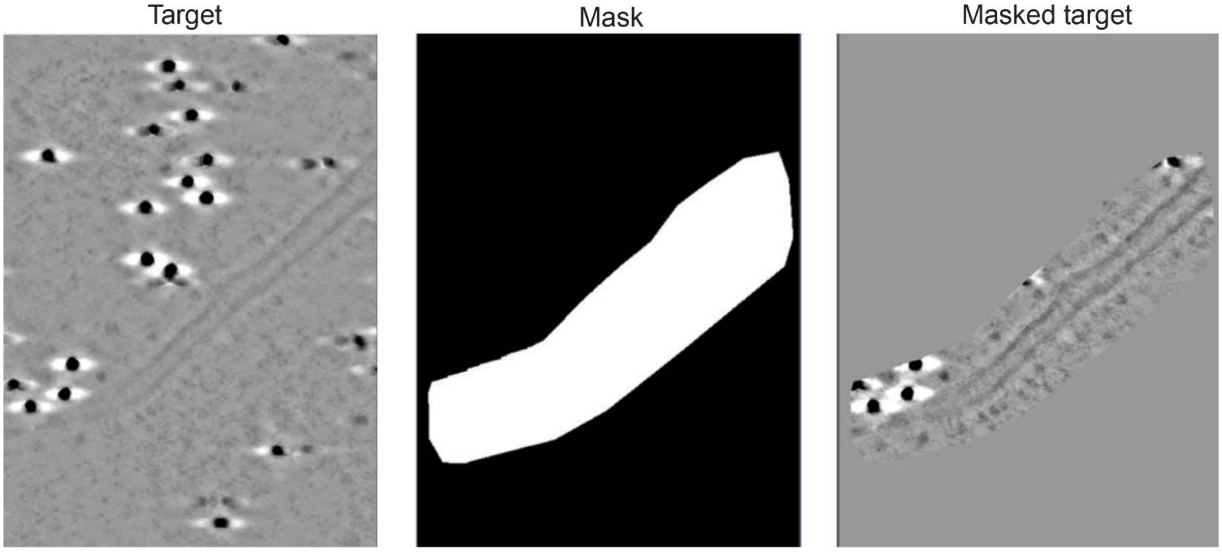

Figure S5 (a) A slice through a tomogram, a binary mask and their multiplication resulting in a masked target image for targeted centroid extraction. (b) A slice through the same tomogram as in Figure 4A, but highlighting coordinates identified by template matching.

Table S1

TS name	Num particles
TS_02	166
TS_03	142
TS_12	141
TS_13	124
TS_15	149
TS_17	89
TS_18	141
TS_19	119
TS_20	110
TS_21	149
TS_22	134
TS_24	152
TS_25	124
TS_26	132
TS_28	141
TS_29	160
TS_31	91
TS_33	122
TS_34	96
TS_35	126
TS_36	113
TS_37	153
TS_38	107
TS_39	112
TS_40	136
TS_41	132
TS_43	146
TS_50	118
TS_51	87
TS_58	95
TS_61	124
**Total particles**	**3931**
